# A Single-use Strategy to Enable Manufacturing of Affordable Biologics

**DOI:** 10.1016/j.csbj.2016.06.007

**Published:** 2016-07-05

**Authors:** Renaud Jacquemart, Melissa Vandersluis, Mochao Zhao, Karan Sukhija, Navneet Sidhu, Jim Stout

**Affiliations:** Natrix Separations Inc., 5295 John Lucas Drive, Burlington, Ontario L7L 6A8, Canada

**Keywords:** DSP, downstream process, USP, upstream process, CoG, cost of goods, OpEx, operating expense, CapEx, capital expense, cGMP, current good manufacturing practice, HCP, host cell protein, EBA, expanded bed adsorption, SMB, simulated moving bed, MV, membrane volume, FT, flow through, B&E, bind and elute, PAT, process analytical technology, FDA, Food and Drug Administration, EMA, European Medicines Agency, Flexible, single-use facilities, Continuous bioprocessing, Antibody manufacturing paradigms, Process economics, Affinity membrane chromatography

## Abstract

The current processing paradigm of large manufacturing facilities dedicated to single product production is no longer an effective approach for best manufacturing practices. Increasing competition for new indications and the launch of biosimilars for the monoclonal antibody market have put pressure on manufacturers to produce at lower cost. Single-use technologies and continuous upstream processes have proven to be cost-efficient options to increase biomass production but as of today the adoption has been only minimal for the purification operations, partly due to concerns related to cost and scale-up. This review summarizes how a single-use holistic process and facility strategy can overcome scale limitations and enable cost-efficient manufacturing to support the growing demand for affordable biologics. Technologies enabling high productivity, right-sized, small footprint, continuous, and automated upstream and downstream operations are evaluated in order to propose a concept for the flexible facility of the future.

## 1 Introduction

### 1.1 Expensive Biologics

In the U.S. alone, biologics account for 40% of prescription drug spending, despite only 2% of the population using biologic drugs [1]. The cost of these medications is quickly rising from expensive to simply unaffordable. This global issue will likely become more problematic with the steady growth of the world population and the advent of new biologic therapies. Some strategic measures have been implemented to mediate the rising prices such as a cost-effectiveness analysis methodology which appraises health interventions and selects technologies based on the returns expected for a financial investment, therefore urging pharmaceutical companies to provide biologics at competitive rates [2]. Many governments have passed legislature aimed at reducing expenditures on follow-on biologics such as biosimilars [3] which decreases the costs of a product entering the market by shortening the approval pathway, ensuring compulsory licensing and encouraging data sharing. These strategic initiatives are expected to moderate the high costs of new biologics by encouraging a competitive market. Despite these strategic initiatives, drug prices are reaching as high as $50,000 per treatment [4]. At the same time, innovation manufacturers (bringing a new drug to market) are battling low profit margins. The average total cost of launching a novel drug was $3 billion between 2004 and 2009, but the R&D portion of that total rose from between 18% and 23% to 34%. Expenses exceeded sales of novel drugs in this five year period [5].

### 1.2 Manufacturing Status Quo

Although monoclonal antibody (mAb) production has experienced improvements from some single-use (SU) upstream and downstream technologies, holistic strategies are required to combine and implement these advances for more efficient and economical production. Typical mAb manufacturing practices involve several stages of inoculum development in small reactors followed by cell cultivation in larger stainless steel bioreactors (5000 L to 25,000 L). The WAVE bioreactors, now offered by GE Healthcare, were the first SU bioreactors designed for large scale manufacturing. Introduced in 1996, the WAVE bioreactor consisted of a plastic bag on rocker platform that provides agitation and gas transfer. Despite the evolution over the years, the WAVE bioreactor is only available in volume sizes of up to 500 L and therefore usually operated as part of seed expansion in mammalian cell culture-based bio-therapeutics manufacturing [6].

SU stirred tank bioreactors, such as GE Healthcare's Xcellerex bioreactors that were introduced in the mid- to late 2000s, are now commonly operated in small- to mid-scale bioprocessing projects [7]. In this reactor, cells are cultured in a replaceable plastic bag that is housed within a stainless steel tank. Mechanical agitation is supported through the bottom, center-mounted magnetic drive that couples with the impeller in the bag [8].

Due to weight limitations, SU stirred tank bioreactors above 2000 L are not feasible. However, over the last decade, there has been a dramatic increase in expression yield from progress in cell lines, expression systems, and culture [7]. These advances have made the large scale bio-manufacturing with the Xcellerex bioreactors more practical. For instance, 100 kg/year of mAb can be manufactured in a few SU 2000 L bioreactors operated continuously or in parallel, whereas multiple ≥ 10,000 L bioreactors were required for the same output only a decade ago.

Bioreactors have seen dramatic growth in the U.S. SU systems market for cGMP (current good manufacturing practice) manufacturing, and this increased adoption is expected to continue. In a 2014 survey conducted by BioPlan Associates, 65.6% of clinical scale manufacturers and 42% of commercial scale manufacturers have cited implementation of SU bioreactors for new facilities as a major factor that have resulted in improvements in their bioprocessing [7]. According to the same report, the SU upstream bioprocessing market is expected to grow by > 320% in five years. Following cell culture, primary recovery is performed by centrifugation and filtration from which the cell harvest is passed along to low productivity downstream processes (DSPs) [9]. In large manufacturing facilities, multiple bioreactors supply one or more purification trains. These purification trains have evolved to a common platform over years, starting with Protein A affinity chromatography and followed by polishing steps (usually anion and cation exchange chromatography and sometimes hydrophobic interaction chromatography in place of cation exchange), and virus inactivation and filtration [10]. These long, complicated processes result in non-optimal yields and increased risk of lost batches due to contamination or operator error. Advances in cell culture technology have increased mAb titers, but manufacturers cannot take advantage of high productivity bioreactors until the purification bottleneck has been mitigated.

Cost of goods (CoG) related to manufacturing has been identified as one of the main cost-drivers of expensive biologics [11] with Protein A resins being among the highest cost consumables in mAb processing (up to €14,000/L) [12]. Traditional industrial mAb processes requiring large volume unit operations, have high operating expenses (OpEx) influenced by expensive chromatography resins and large buffer volumes. Manufacturing facilities also necessitate high capital expenses (CapEx) due to constructing cGMP facilities, stainless steel reactors, large filtration and chromatography skids, as well as associated piping and hardware for the entire 20 + step process [9].

### 1.3 The Solution Resides in Innovation

Increased competition from biosimilars and other follow-on biologics is forcing established manufacturers to find or develop and integrate new manufacturing technologies to stay competitive and conserve their market share. The current manufacturing paradigm of large scale cGMP manufacturing operations is no longer needed for the majority of biologics produced today. Technology advancements in cell culture operations and equipment, purification media, techniques, and hardware have simplified the large stainless steel operations into manageable, small operations with disposable technology. A comparison of data from typical processes from 1982 and 2004 show rises in stirred-tank product titers from 50 mg/L to 4700 mg/L, with approximately 8 × improvement in specific productivity [13]. Sophisticated SU technologies such as perfusion bioreactors are pushing productivities that are more than 25 × higher than batch culture [14] and emerging downstream technologies, such as membrane chromatography, have demonstrated potential to match the high bioreactor throughputs. Affinity chromatography is the purification method of choice for antibodies because of the highly specific binding between Protein A ligands and the Fc region of immunoglobulins [15], but productivity is limited by the slow flow rates of resin columns. A novel membrane affinity technology offers high yield and > 99% purity in a one-step purification process with much faster flow rates than resin columns [16], [17]. With many improved technologies emerging, the stage is now set for the industry to pragmatically combine such technical innovations in bioprocessing to reduce the costs of biotherapeutics.

In this paper, we present innovative concepts facilitated by modern upstream and downstream processing technologies that enable manufacturing of affordable biologics, with special emphasis on purification technologies. These novel strategies increase flexibility and overall output, decrease manufacturing CoG, and reduce facility footprint. The integrated facility design presented in this paper also enables local manufacturing, further resolving problematic inventory and supply chain issues faced by the pharmaceutical industry today.

## 2 Proposed Engineering Concept: Flexible Facilities of the Future

### 2.1 Industrial Production at Lab Scale to Reduce Costs and Increase Flexibility

The current standard manufacturing scheme for biologics is optimized for fed-batch bioreactors that are well adapted for suspension cell culture processes, followed by a combination of different filtration and chromatography unit operations to achieve the target purity requirement and yield. Both fed-batch bioreactors and resin column chromatography operations experience an over-sizing problem when scaled up to pilot and manufacturing process scales. Fed-batch reactors, which comprise about 90% of commercial biologics production, require large-volume tanks to counter the low productivity per unit volume [18] and resin chromatography columns, limited by intrinsically slow mass transfer rates, have to be over-sized in order to operate at a higher flow rate to achieve desirable productivity [19]. This approach of attaining better productivity via enlarged working units occurs at the expense of process economics and flexibility. Large reactors (up to 25,000 L) and chromatography columns (up to 2 m in diameter with 10 cm to 20 cm bed height) directly increase the facility cost due to increased building and equipment expenses as well as associated piping and hardware costs (including preparing, holding, and cleaning) [20].

In addition to the economic disadvantage, over-sized bioprocess plants offer limited flexibility, which directly contradicts the emerging trend in the biopharmaceutical industry of production on demand [21], [22]. In the case of low demand (for example, GSK reported Cervarix sales were down 27% in Q1, 2015 [23]), process capacity will be greatly wasted; therefore the unpredictability of demand necessitates facility flexibility. Moreover, the industry is transitioning from large, single-product facilities to scalable, multi-product facilities for greater product variety [24]. The emergence of personalized medicine and battlefield medicine as well as smaller product campaigns associated with orphan drugs, biomarkers, and smaller disease paradigms puts more pressure on manufacturing volume management [25], [26], [27]. Multi-product, small-volume capabilities are unrealistic for over-sized bioprocess plants because of rigid infrastructure and non-disposable technologies. In these situations, modification of the facility to adapt to a more flexible design is tenable, but at a significant cost and at a facility operation that is only marginal in flexibility.

**Fig. 1:**
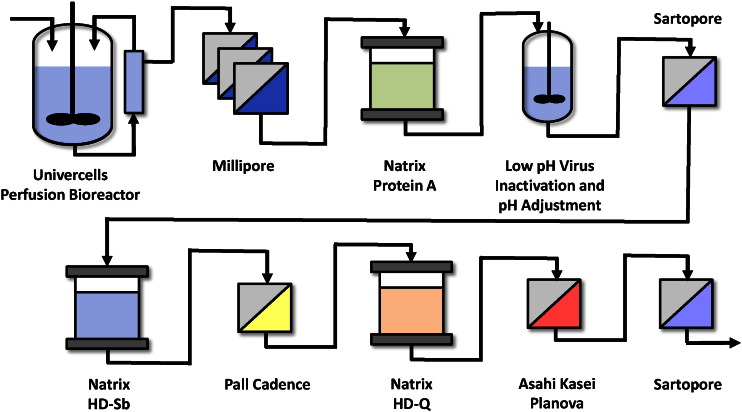
Univercells-Natrix automated, integrated, quasi-continuous mAb process concept. The continuous perfusion bioreactor and state-of-the-art DSP techniques are combined for optimum productivity in a small, contained operation.

Many advanced technologies have been explored to achieve full capacity utilization with more flexibility at less capital expenditure. By integrating state-of-the-art techniques and equipment in upstream and downstream operations, manufacturing processes can be right-sized, creating a smaller footprint which enables facilities to add or remove unit operations depending on the demand without wasted capacity and extra expenses. For example, Fig. 1 presents a concept developed by Univercells and Natrix Separations for a biosimilar mAb. It is based on standard purification architecture but with emerging technologies to enable a much simpler, quasi-continuous platform. The advantages of this process can be pushed even further when improved process architecture is incorporated, as demonstrated later.

#### 2.1.1 Right-sizing Upstream Processes

For an upstream process using fed-batch cell culture, process scale-up can be achieved by a faster cell culture production rate using multiple bioreactors that are harvested at a certain frequency per week (“run rate”), but this puts strain on the purification process or requires larger or dual column/filtration operations to accommodate the biomass harvest frequency. A more common scale-up method to increase manufacturing capability is via larger working vessels, which requires developing a dedicated bioprocess facility through expansion or new construction [28]. For example, for commercial scale mAb production, the traditional fed-batch bioreactor usually cultures cells in 10,000–25,000 L stainless steel tanks for 7–21 days with a product yield of 2–6 g/L [18]. The renewed interest in perfusion bioreactors together with cell culture advancements such as high titer mammalian cell lines, transgenic expression, and microbial expression can significantly improve the upstream productivity per unit volume and reduce the requirement of high-volume units [29]. Perfusion reactors utilize a continuous supply of cell culture media while the growth-inhibiting by-products are constantly removed over a prolonged production phase (typically > 20 days) to achieve 10–30 times higher cell density compared to a fed-batch reactor [30]. Longer run duration and the ability to sustain biomass levels enable perfusion bioreactors to offer a productivity advantage (in terms of mg/L/d) of at least 4-fold as compared to a fed-batch unit with the same reactor volume [18]; therefore, the same product quantity can be produced with less space and capital cost. The continuous nature of a perfusion operation also makes it a great candidate for continuous processing of biopharmaceutical proteins. Implementation of perfusion reactors has been successfully commercialized, ranging from large biopharmaceutical companies such as Pfizer, Genentech, Shire, and Genzyme/Sanofi [31], [32], [33] to small companies and innovative vaccine manufacturers such as CMC Biologics and Crucell [34], [35]. Although there are still drawbacks to the technology such as usage of large volumes of medium, and high level of operator training required due to the complexity and intensity of the operation, the economic gain from smaller vessels and facilities has the critical impact on process considerations [18], [36].

#### 2.1.2 Right-sizing Downstream Processes

In downstream processing, clarification, capture, and polish steps can be optimized by using high throughput, SU, and continuous technology. For clarification, centrifuges are not the right solution and are becoming outdated because they are difficult to scale and complicated to operate [37]. Filtration is an alternative to centrifugation due to much simpler implementation in flexible, SU, continuous processes and has been demonstrated successfully for large scale, commercial processing (Humira®), but the original designs in stainless steel housings were complex at large scale. The new disposable filtration designs offer more flexibility and scalability. For example, the Stax disposable depth filter system (Pall) is a versatile, robust platform that can be operated in different modes depending on the process [38]. Millipore's Clarisolve as well as D0HC and X0HC adsorptive depth filters can be used for primary or secondary clarification directly from the bioreactor or after low pH precipitation of impurities. By reducing host cell protein (HCP) and DNA, these depth filters maximize loading on chromatography columns while eliminating cell debris [39]. Filter aids like diatomaceous earth can be added to cell culture fluid to prevent blockages in filters therefore allowing large batches to be clarified with maximum efficiency in SU formats as demonstrated by Sartoclear Dynamics (Sartorius Stedim Biotech) [40]. These new modalities offer adaptability to various process scales as well as contaminant reduction which consequently helps to maximize the binding capacity of capture chromatography.

Different chromatography techniques (affinity, ion exchange, hydrophobic interaction) have been widely implemented in biologics purification in the format of resin columns. The majority of functional ligands are grafted within internal pores of polymeric chromatography resins. In order to interact with the ligands, molecules have to take long and restricted diffusion pathways which significantly hinder mass transfer and limit flow rate [19]. In the attempt to increase productivity, resin columns are sized by volumetric flow rate instead of capacity, resulting in over-sized unit operations (up to 2 m in diameter with 10 cm to 20 cm bed height) that require vast investment in large columns (especially for costly Protein A resins for mAb capture), associated hardware, supporting systems, and facilities. To be cost effective, these large columns need to be amortized over many cycles and batches, which increase the oversight of quality and regulatory groups for consistent processing. Besides the economic issues, large columns can suffer from scale-related packing problems including hysteresis, edge effects, and resin compression [20]. Alternative formats for purification units including expanded bed adsorption, simulated moving beds, and membrane chromatography are gaining popularity.

Expanded bed adsorption (EBA) is a potential cost-, time-, and space-saving technology because it integrates solid–liquid separation and adsorption, therefore reducing several unit operations (filtration, centrifugation, chromatography capture) to one step. EBA can process high cell density feeds directly from a bioreactor without risk of fouling because the fluidized bed increases the interstitial volume between adsorbent particles therefore allowing cell components to flow through [41]. While EBA offers obvious advantages such as reducing CapEx, buffer volumes, and operation time, there can be issues such as the need for recirculation and non-specific adsorption which affects bed stability and purification performance [42]. New advancements to EBA such as the second generation EBA Rhobust Technology provide an improved, right-sized solution for mAb capture. Data presented at the BioProcess International Conference and Exposition shows that in comparison to a packed bed Protein A column, Rhobust MabDirect Protein A requires one third of the processing time, half of the buffer usage, offers 12% better yield, improves DNA clearance, and has comparable purity [43]. As bioreactor cell density and productivity increase, EBA is a promising option that improves throughput and eliminates the need for large filtration areas required to deal with fouling from crude feeds.

Simulated moving bed (SMB) technology presents a fully continuous method for performing chromatography. The BioSMB system built by Tarpon Biosystems Inc. (now Pall) allows continuous loading of feed as well as continuous elution as multiple Protein A columns are cycled through the load, wash, and elution stages at different times [44]. The Accelerated Seamless Antibody Purification (ASAP) process is a fully disposable, continuous mAb DSP, based on AKTA periodic counter-current chromatography (PCC), including Protein A, mixed mode, and anion exchange resin columns where the three columns are cycled simultaneously [45]. Another advantage of SMB mode is that columns can be connected in series so that any breakthrough from the first column is loaded onto the second column allowing the entire capacity of the first column to be used without losing valuable product [46]. This allows the use of columns with shorter bed heights that may have shallower breakthrough curves, but can operate at faster residence times which overall increases productivity [47]. Compared to batch resin chromatography, SMB provides advantages including 30% high productivity, up to 40% increase in loading capacity, and up to 27% less buffer consumption [46]. Executing chromatography steps in continuous SMB mode not only decreases risk of contamination from human error and stoppages in the process, but also decreases operation costs by reducing the amount of resin, buffer, and time required for processing [48], [49], [50], [51].

An alternative to resin columns is nanofiber adsorbents such as Puridify's FibroSelect platform, which, although it has low binding capacities (10 mg/mL), is able to flow at very fast flow rates (2400 cm/h) enabling high productivity as well as continuous processing [52]. Monolithic platforms would also be good for mAb purification because their high porosity structure provides effective mass transfer for the antibody-sized targets and the ease of material preparation reduces manufacturing costs, but monolith operation has yet to be translated from analytical scales to industrial scales [53]. Monoliths are commercially available from several manufacturers including BIA Separations, Millipore, and Sepragen [53].

Another alternative to resin chromatography is membranes which have been gaining popularity for their ease of use and high throughput. Unlike resin beads that heavily rely on diffusion, mass transport through membranes is dominated by advection which allows much higher flow rates. Conventional ion exchange membranes are available from several suppliers including Pall (Mustang) and Sartorius (Sartobind), and these are well suited for flow through (FT) applications. However, since the ligand density is lower than on resin beads, bind and elute (B&E) applications are limited [54]. Natrix's HD membrane technology contains a high density of binding ligands in porous polymer hydrogels which allows significantly improved flow characteristics without compromising binding capacity. According to mAb process simulations, cycling smaller devices with high binding capacity significantly reduces CapEx because required hardware is expensive for large columns, and decreases OpEx due to the high cost of media (especially Protein A) [12]. With this new chromatography media, higher throughput can be achieved without over-sizing the device and rapid cycling can be utilized to reduce media volume, leading to increased productivity and flexibility at lower CapEx.

### 2.2 Integrated Facility Concept: Automated, Continuous, Small Footprint Antibody Production

**Fig. 2:**
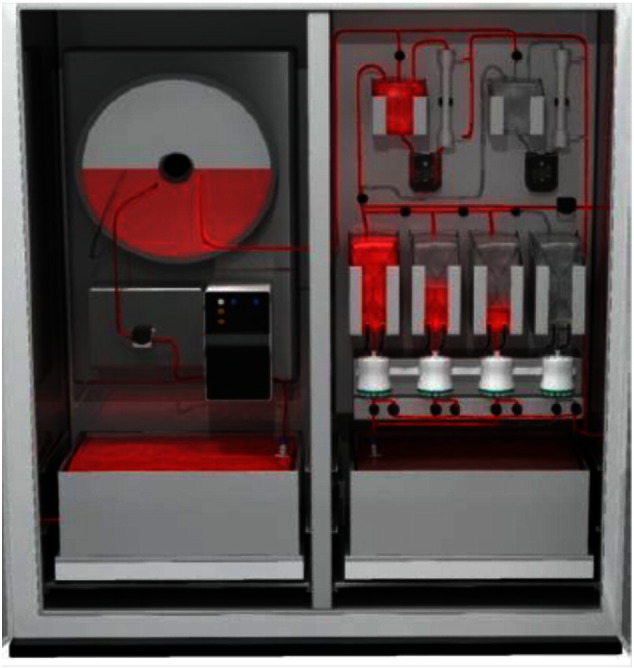
Univercells' modular concept for combining USP and DSP in a small-footprint cabinet. The output from the high-productivity perfusion bioreactor (in the left chamber) is continuously feeding into the purification train (in the right chamber). The size and productivity of the perfusion bioreactor is matched with the downstream recovery process for efficient biologic manufacturing.

State-of-the-art technologies for upstream processes (USP) and DSP described above can be integrated into a matched process, well-balanced between the bioreactor output and the purification throughput. In theory, the entire process can be contained in a single, small footprint cabinet, as shown in Fig. 2. The concept is based on standard mAb purification architecture where high throughput of USP and DSP enables production in approximately 20 ft^2^ of the GMP suite, which could be itself a modular clean room [35]. One cycle of the DSP, including capture, virus inactivation, polishing, and sterile filtration is designed to be completed in only 24 h.

**Fig. 3:**
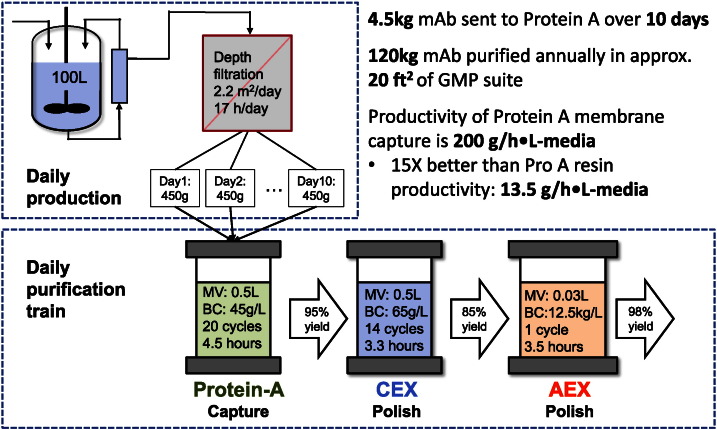
Rapid multi-cycling enables small-footprint DSP to match the throughput of high-productivity USP. The conceptual process projected from lab scale proof of concept demonstrates a purification train that is capable of keeping pace with the perfusion bioreactor output. The process is made up of Natrix membrane columns (Protein A, HD-Sb (CEX) and HD-Q (AEX)) that are sized just right for productivity, economy, and flexibility.

This strategy is depicted in Fig. 3, where a detailed process flow diagram derived from Fig. 1 shows the operation parameters (extrapolated from lab scale proof of concept) of daily production and purification. For this hypothetical process, a 100 L high density perfusion bioreactor can achieve higher product output compared to the traditional fed-batch cell culture technique. Operating at 2 volumes per day with a titer of 2.5 g/L, the perfusion bioreactor can produce up to 5 kg of mAb over 10 days (approximately 120 kg annually over 24 perfusion runs). A novel, SU perfusion reactor in development by Univercells has the potential to realize this high efficiency production [55], [56]. In contrast, producing 100 kg of mAb annually using fed-batch bioreactors typically requires 15 batch runs with a titer of 5 g/L in reactors 20 × larger [57].

Hydrogel membranes that are appropriately sized for large-scale mAb purification are a cost efficient solution for keeping up with high culture productivity as shown in Fig. 3 (data shown for each membrane column is extrapolated from > 3 independent lab scale experiments). A prototype Protein A affinity membrane column has demonstrated 45 g/L binding capacity with 6 s residence time while still achieving over 95% recovery. A 0.5 L membrane column would be cycled 20 times per day (total 4.5 h) to process 4.5 kg of mAb over 10 days. In comparison, a much larger 24 L resin column would take 5 cycles to process the same batch in a similar time frame. This example shows how right-sizing promotes savings because the large resin column uses only a fraction of its capacity per batch.

To continue the high productivity purification train, membrane columns are also used for polishing. Fig. 3 shows Natrix's HD-Sb followed by HD-Q, each having 6 s residence times as well. The HD-Sb membrane requires only 14 cycles (total 3.3 h) per day to process the eluate from the Protein A affinity membrane. The high binding capacity of HD-Q can process the HD-Sb purified product with only a single cycle in FT mode for final impurity reduction. Over the course of 10 days the media life of these membranes is expended to process the entire batch and capacity is not wasted to enable the fast flowrates. These small membrane columns (relative to resin columns), allow large quantities of mAb to be processed in a small footprint facility.

## 3 High Productivity, Single-use Membranes: Lab Scale Data Proof of Concept

### 3.1 Materials and Methods

A new Protein A membrane formulation is being investigated at Natrix Separations. The new membrane is composed of a base formulation that can be coupled in a secondary reaction to an affinity ligand. The ligand chosen for these studies is a base-stable Protein A sourced from a known vendor.

The Protein A affinity membrane experiments were run on Natrix prototype membranes using four clarified innovator and biosimilar antibodies (mAb1, mAb2, mAb3, and mAb4) from Chinese Hamster Ovary (CHO) cell culture. MAb1 and mAb3 were Human IgG1 antibodies with molecular weight 144,190 Da and pI 8.25. MAb4 was Human IgG1 antibody with molecular weight 145,531 Da and pI 8.45 and mAb2 was obtained in confidence from a biopharmaceutical company for membrane studies. Prior to loading on the membranes, mAb1 and mAb2 were pre-treated with Millipore X0HC depth filtration, whereas mAb3 and mAb4 were filtered with 0.2 μm bottle top filters. Further information on buffer systems and purification devices are detailed below, along with the testing results. Cleaning treatment for the Protein A membrane following elution is 0.1 M acetic acid strip and 0.1 M sodium hydroxide cleaning in place (CIP).

Protein A membrane lab scale experiments for evaluation of HCP clearance for various mAbs and buffer conditions were performed with 1 layer of membrane assembled in a 25 mm diameter stainless steel housing (MV = 0.12 mL). The reference resin purification platform process included Amsphere™ Protein A column (ID 50 mm, bed height 20.5 cm, bed volume 403 mL) followed by CEX purification using POROS XS column (ID 50 mm, bed height 23.5 cm, bed volume 461 mL) followed by AEX purification using HiTrap™ QFF column (5 mL bed volume). For the experimental membrane platform process, Protein A purification was done using 2 layers of membrane stacked in a 47 mm diameter test cell (MV = 0.8 mL) followed by CEX purification using Natrix HD-Sb Recon (MV = 0.87 mL) followed by AEX purification using Natrix HD-Q membrane (MV = 0.04 mL). All experiments were performed on a GE Healthcare AKTA Purifier.

### 3.2 Results

#### 3.2.1 Membrane HCP Clearance Equal to Resins for Various mAbs and Loading Conditions

In this study, SU Protein A membrane and Protein A resin (Amsphere™ Protein A column, ID 5 mm, bed height 50 mm, bed volume 1.0 mL) were tested at laboratory scale with four different mAbs to compare the performance of the different media (Table 1) and assess platformability of the technology. Based on the data in Table 1, the level of eluate HCP from the Protein A membrane compared very well or even better than the Protein A resin, even with a wide HCP feed complexity, from > 25,000 ppm to > 1,400,000 ppm (as determined by the Cygnus III HCP ELISA kit). The high flow rate of membrane columns (6 s residence time) allows purification of similar loads in only a fraction of the time compared to resin columns (4 min residence time).

The Protein A membrane was further tested with three different buffer systems (Table 2) using mAb2, mAb3, and mAb4 to compare HCP reduction performance under different conditions (Table 3). Different mAb species can have varying characteristics and therefore HCP reduction and elution efficiency can be improved by adjusting the buffer strength, salt concentration, and pH as shown in Table 3. The high flowrate used with membrane columns allows for a fast elution step; therefore very low pH (pH 3.0) can be used to quickly elute the column without causing aggregation. The data obtained support a robust impurity clearance for different mAbs and matrices across multiple buffer systems. In summary, the combined data from Table 1, Table 3, reveal the Protein A membrane can support a wide range of mAbs, complex feed impurities, and an array of buffer conditions with great HCP reduction to offer a flexible capture option that can adapt to varying feed and process conditions.

#### 3.2.2 Fully Single-use Downstream Process

The Protein A membrane improves mAb capture, and similar advancements have been reported for membrane columns used in polish applications [58], [59], [60], [61]. A lab scale process comparison between a fully SU membrane column platform and a traditional resin column platform was performed using mAb3 to demonstrate the advantages of membrane columns (Table 4). Three chromatography steps for typical mAb purification (Protein A, cation exchange, and anion exchange) were studied at lab scale for both media. The membrane process showed comparable HCP clearance for the cation exchange and anion exchange steps and significantly better clearance for the Protein A step (see Table 4). The yields were similar for both processes, however the membrane columns were able to maintain equal or better HCP reduction & yield with faster flow rates and higher binding capacities. This translates to a much higher productivity per volume of media, resulting in a higher throughput from the membrane process with similar purity to the traditional resin column process.

#### 3.2.3 Two Flow Through Steps to Optimize Downstream Throughput

The productivity of the polish processes discussed above can be further increased by using the cation exchange membrane (HD-Sb) column in FT mode instead of B&E mode (Table 5). In FT mode, the mAb throughput capacity is 300 g/L with very good HCP reduction, therefore increasing the amount of feed that can be processed in one cycle. Table 5 shows examples of 2 different processes using HD-Sb in FT mode followed by HD-Q in FT. Different Protein A purified mAb feeds are used, with the feed for process 1 having high aggregate levels and the feed for process 2 having greater HCP concentration. For both processes 2 coupons of the corresponding membrane chemistry were layered in a 25 mm diameter test cell. Buffer compositions are listed in Table 6. The HD-Sb chemistry, with comparable performance at pH 5.5 and pH 7.5 in the FT mode (Table 5), demonstrates the robustness of Natrix HD membranes. One notable performance advantage of the HD-Sb membrane is the ability to reduce high aggregate levels in the flow through mode (Table 4) as well as B&E mode (data not shown). Further, at pH 7.5, the FT conditions are the same for the HD-Sb and HD-Q steps, creating the strategic option of a directly coupled tandem polish process, which eliminates the need for buffer adjustment between unit operations. Therefore, FT mode at pH 7.5 simplifies the process and reduces processing time and expense. Overall, the demonstrated process performance of the HD-Sb and HD-Q membranes allow a wide range of process operations that can significantly increase process productivity while maintaining consistent impurity reduction and good yield.

## 4 Impact of Single-use Strategy on Manufacturing Cost of Goods

Integrating innovative tools for both upstream and downstream processing, using a holistic approach for the process and facility, provides an opportunity for complementary technologies to create synergistic manufacturing strategies [62]. The shift in biopharmaceutical manufacturing towards more flexible, small footprint facilities requires significant changes to many traditional processes. Many of the legacy facilities are often operating at partial capacity with outdated processes, sometimes using equipment with dated engineering designs and/or limited flexibility. These facilities will have trouble competing with biosimilars as well as keeping up with new FDA (Food and Drug Administration) and EMA (European Medicines Agency) regulations due to the high capital expenses, operational costs and regulatory burden [63], [64].

On the other hand, SU systems are increasing in popularity, as a survey of biopharmaceutical manufacturers' reports, for various reasons including reduced capital investment, decreased risk of product cross-contamination, reduced start-up time, and eliminating cleaning requirements [65]. Simulation and economic modeling software used in the biopharmaceutical field, such as Biosolve Process (Biopharm Services) or SuperPro Designer (Intelligen) [66], are able to evaluate different manufacturing scenarios to determine the impact of SU strategies on process economics. An extensive review on the economics of mAb manufacturing is hindered by the lack of peer reviewed articles because many manufacturers do not share this sensitive information except at conferences. This section summarizes data from modeling software and existing facilities that has been presented at conferences or published in peer reviewed journals.

To enable more affordable, higher throughput processing, existing facilities can be retrofitted to incorporate advanced SU technologies. With logistics, staff, and capital investment already in place as well as reduced regulatory obstacles, established plants can smoothly transition to more economical processing. Biosolve analysis shows that operating costs per gram of mAb for a SU facility compared to a stainless steel facility are 22% lower and this is primarily due to less labor, utilities, maintenance, and waste [67]. In a case study presented at Cambridge Healthtech Institute's PepTalk conference, annual savings of $250,000 in WFI (water for injection) generation costs and $60,000 in labor time for set-up and cleaning stainless steel tanks were realized in a clinical plant that was retrofitted to implement disposable bulk freeze containers and buffer hold bags [68]. When looking at DSP improvements, chromatography resins make up a large portion of the costs so changing a polishing step from resin chromatography to a SU membrane column can significantly reduce expenses, as demonstrated by a Biosolve Process model that evaluates commercial mAb production [69]. In this study of 1000 L and 5000 L scales and various mAb titres, the unit operation cost is 19% to 33% lower for the membrane process and buffer volume is decreased by up to 55% [69].

Instead of retrofitting an existing facility, new-build, state-of-the-art SU facilities offer the same advantages and more without space constraints, non-optimized layouts, difficult installations, and scheduling constraints [70]. While an initial capital investment is still required, this cost is much lower compared to building a multi-use (MU) facility because many stainless steel skids and their associated hardware are eliminated, equipment is smaller, and the building is smaller. In a whitepaper published by Biopharm Services Limited, modeling software shows that building a large-scale mAb process with thirty 2000 L disposable bioreactors has a total capital investment of $250 M which is a significant reduction from the $352 M capital investment required for a stainless steel facility with the same capacity [71]. In another case study that compares the costs for a SU versus MU 2 × 1000 L new facility, the SU facility saves €11 M annually in capital investment and only costs €1 M more in operating costs [72], [68]. This model shows that significant CapEx reductions result from lower engineering costs (decreased by 83%) and instrumentation costs (decreased by 37%) and the increased running cost was influenced mainly by a higher consumables cost (increased by 51%) [72].

In addition to converting facilities to SU operations, the clean room environment can be collapsed to encompass individual units which are connected by SU tubing sets as in the FlexFactory concept developed by Xcellerex [73]. This innovative design reduces the amount of space required as well as protects the product from operator contamination. Information shared at the International Society for Pharmaceutical Engineering (ISPE) Strasbourg conference demonstrates that when comparing 2000 L mAb production in a FlexFactory facility design versus traditional infrastructure, the space required is reduced by 45%, water usage is reduced by 87%, and capital cost goes down by 67% [74]. Combined, these improvements cause a 32% reduction in the CoG to $104.8 per gram of mAb [74]. The flexible facilities of the future using self-contained modules are also an attractive option for retrofitting or new build facilities because the modules can be built offsite and then installed in unclassified spaces. Entire processes can be designed in a modular manner which allows fast replication of processes so they can be installed anywhere in the world, as well as easy scale up by just increasing the number of modules [75]. Models for this SU strategy support the same order of cost improvements that was seen with the Xcellerex FlexFactories for an expected capital cost of $25 M for a new-build plant and CoG of mAb in the range of $85/g.

SU process analytical technology (PAT), such as disposable real time monitoring systems, are also gaining popularity. Many bio-manufacturers are adopting real-time SU monitoring of key parameters such as temperature, pH, dissolved oxygen (DO), and biomass. Benefits of disposable monitoring technologies include lack of sterilization requirements, lowered risk of contamination, reduced operator exposure to biohazardous substances, and confirmation of real time process conditions. Examples of disposable sensors include the SU, pre-calibrated SciPress, SciCon and SciTemp sensors offered by SciLog Bioprocessing Systems for in-line pressure, conductivity and temperature monitoring.

Suppliers also offer SU bioprocessing devices fitted with disposable sensors. Sartorius Stedim Biotech, for instance, provides the LevMixer®, a magnetic mixer for volumes between 50 L to 1000 L with pre-assembled SU probes for inline pH and temperature monitoring. Typical applications of the LevMixer include buffer, media and feed preparation, dilutions and pH adjustments, low pH viral inactivation, and product reformulation. Sartorius Stedim Biotech also offers the Flexsafe bioreactors with welded BioPAT ViaMass sensor discs. The SU BioPAT ViaMass sensor, developed by ABER Instruments, detects cell density level in cell cultures through RF impedance spectroscopy.

SU bioprocessing analytical tools are not discussed in depth here since this topic is out of the scope of this article. Readers are encouraged to read (A Biopharmaceutical Industry Perspective on Single-Use Sensors for Biological Process Applications, 2015) for a detailed review.

Although SU technologies bring several key advantages, there are a few important limitations that cannot be overlooked.

First of all, SU tools are often manufactured from plastic derivatives. There have been concerns raised regarding the effect of extractable and leachable compounds, such as antioxidants, plasticisers, and curing agents, from these plastic disposable technologies on the quality of the final product. For example, an extensive study by Amgen reports that bis(2,4-di-tert-butylphenyl)phosphate (bDtBPP), a cytotoxic compound, leaches from certain SU bags and impedes cell growth under many cell culture conditions [76]. The Bio-Process Systems Alliance (BPSA) has issued a comprehensive report outlining recommendations for extractables and leachables testing from SU equipment [77]. On top of the validation concern regarding leachable and extractable materials, the use of plastic SU products also triggers the debate over their potential environmental impact.

The growing implementation of SU products is accompanied by increasing concern about the adverse environmental effects from the regular, large-volume disposal of plastic waste. Some suppliers have assessed the possibility of recycling but concluded that the likelihood is very limited due to mixed plastic content and possible requirement of pre-treating biohazardous materials; so traditional methods of disposing SU plastics through landfill and incineration will remain as the standard [78]. While plastic waste management is a drawback for SU technologies, several studies have shown that the environmental benefit of using SU technology due to reduced consumption of energy, water, and cleaning agents compared to multi-use stainless steel facilities outweighs the disadvantage and can significantly reduce the overall carbon footprint [79], [78].

Consistency and availability of SU materials is another issue that can directly affect the supply of SU products. Biopharmaceutical manufacturers expect availability of manufacturing materials with consistent quality for the duration of the manufacturing campaign. Often, the same material is required for future manufacturing campaigns to maintain consistency and prevent time-to-market delays due to validation and quality assurance conflicts. In order to avoid such problems, suppliers generally maintain a backup supply of materials, and support end-users throughout the product's life-cycle. These issues with SU technologies cannot be overlooked. Yet, the advantages of SU processing such as cost efficiency and flexibility outweigh these potential but preventable problems. Moreover, new high productivity technologies combined in a SU environment are able to provide further improvements to mAb manufacturing. Incorporating high throughput units into one section of a process pipeline can impact other areas such as eliminating the need for extra steps, or allowing downsizing of other unit operations. Not only is production cheaper and less complicated, facility construction and process validation time are significantly shortened. For example, using SU membrane columns instead of MU resin columns in commercial manufacturing eliminates the need for cycling performance studies as well as resin storage and cleaning studies [54].

Disposable technologies were once seen as excellent for multi-product, small volume facilities, but facing technical challenges or limits to their economic advantage at large scales (multi-ton per year), as seen in the case study discussed above (maximum size for SU bioreactors is 2000 L, pre-packed resin columns are limited to 45 cm and recently 60 cm inner diameter) [80]. However, next generation flexible facilities such as the one described in this paper will not suffer from these size constraints since they are based on small batch sizes run multiple times in multiple locations.

Disposable technologies support the shift in biopharmaceutical manufacturing to smaller, flexible facilities for 500 kg or less annual market indications. Although there are still areas for improvement to reduce the CoG for SU manufacturing of blockbuster therapeutics, truly flexible facilities have the potential to be a viable alternative even in the multi-ton market indications.

## 5 Summary and Outlooks

With the growing competition from biosimilars and the shift towards smaller drug paradigms, companies are seeking cost efficient strategies for biotherapeutics manufacturing. The use of innovative technologies enables the optimization of mAb manufacturing in areas such as cost, throughput, and flexibility. Right-sizing unit operations by using improved perfusion bioreactors, depth filtration, and modern chromatographic strategies allow full use of each process operation's capacity as well as downsizing of equipment and related infrastructure. In this article, an in-depth look at platformable chromatography membrane columns for Protein A capture as well as ion exchange polishing demonstrates equal or better performance than traditional resin columns with much higher productivity; a result of high loading capacities and residence times of only 6 s. A conceptual process integrating state-of-the-art perfusion reactors and these SU membrane columns in a small footprint, modular facility can promote much simpler and faster mAb development and production.

Replacing traditional facilities, or first generation flexible facilities where disposable technologies are implemented in the traditional paradigm, with a truly flexible concept design allows for easy and rapid scale up by increasing the number of cost-efficient production units. Industrial production at lab scale no longer requires technical and regulatory scale-up since these production units can be incorporated into modular clean rooms. This simplifies the replication (cloning) of the process such that manufacturing facilities can be installed anywhere in the world in only a few months (in-market/for-market strategy) with no need to redevelop and characterize the process at larger scale. Truly flexible facilities will experience minimum expenses during clinical phases and grow with market demand, hence minimizing risks while optimizing time to market and global distribution without the challenges of complex worldwide distribution logistics.

These changes have begun to be implemented in mAb manufacturing but innovation does not have to stop here. Many areas of biologics production, such as vaccine manufacturing where some current facilities are more than 40–50 years old and use outdated, non-optimized technology [35], can benefit from the cost reductions associated with integrating emerging technologies. Despite the current proofs of concept and successful introduction of new techniques in mAb processes, there may be unforeseen challenges when extending flexible manufacturing strategies to other biologics. For example, perfusion bioreactors may not be able to deliver expected throughputs, challenging feeds may require larger filtration areas [81], and/or new chromatography media might not meet productivity targets; all of which would increase the CoG and facility footprint. Further development of SU, flexible technologies may be needed if expenses become too high or integration and automation cannot be realized.

A final step to high throughput biopharmaceutical production is implementation of state-of-the-art PAT for continuous monitoring and adjustment to ensure specifications are met. Small production skids, disposable units, variable raw materials, and lack of real-time online measurement devices are challenges for PAT integration, but new, sophisticated instruments show promising improvements [82]. Product attribute control is now possible within the bioreactor, for example with MAST (Modular Automated Sampling Technology), and advances in Raman spectroscopy, at-line surface plasmon resonance (SPR), SU sensors, chemometric methods, and ultra high performance liquid chromatography (UPLC) at line monitoring are helping manufacturers overcome hurdles in DSP PAT integration [83], [83], [84], [85], [86], [87], [88].

While there are technologies available for optimizing biologics manufacturing, the industry is slow to move away from the traditional processes that have been proven to work even though they are becoming outdated. It will become necessary for companies to adapt to SU and continuous processing so they can stay competitive in global markets and expand production to developing countries. Continuous processing is also encouraged by the FDA because it reduces manual handling of products, promotes better process control, and is aligned with their quality by design (QbD) initiatives [89], [90], [91]. It is postulated that when combined in a holistic, SU based process and facility strategy, the technology advancements described here will aid in reduction of drug substance manufacturing costs. Furthermore, it will enable a production paradigm that is suitable for local manufacturing needs in developing countries, meeting drug manufacturing requirements and supplying patients with lifesaving medicines.

## Figures and Tables

**Table 1:** Comparison of HCP reduction using Protein A membrane and Protein A resin for four different mAbs. (* pretreated feed). Equilibration/wash 1 buffer: 1 × PBS, pH 7.4. Wash 2 buffer: 1 × PBS + 1 M NaCl, pH 7.0. Elution buffer: 100 mM glycine, pH 3.0.

		HD-A membrane		Protein A resin column	
	Feed HCP (ppm)	Load (mg/mL)	Eluate HCP (ppm)	Load (mg/mL)	Eluate HCP (ppm)
mAb 1*	25,600	30	102	25	203
mAb 2*	89,667	30	307	25	247
mAb 3	319,649	25	527	25	2404
mAb 4	1,417,391	30	1171	25	1123

**Table 2:** Equilibration, wash 1, wash 2, and elution buffers for 3 different buffer systems used to evaluate HCP reduction for three mAb feeds.

	Equilibration and wash 1 buffer	Wash 2 buffer	Elution buffer
Buffer System 1	1 × PBS, pH 7.4	1 × PBS + 1 M NaCl, pH 7.0	20 mM sodium acetate + 50 mM NaCl, pH 3.5
Buffer System 2	20 mM sodium phosphate + 150 mM NaCl, pH 7.6	20 mM sodium phosphate + 1 M NaCl, pH 7.0	100 mM glycine, pH 3.0
Buffer System 3	20 mM Bis-Tris + 20 mM NaCl, pH 7.6	20 mM Bis-Tris + 1 M NaCl, pH 7.0	20 mM Bis-Tris + 20 mM NaCl, pH 3.5 (acetic acid for pH adjustment)

**Table 3:** HCP reduction using Protein A membrane with different buffers and mAbs.

	mAb 2	mAb 3	mAb 4
Feed HCP (ppm)	89,667	285,948	1,417,391
Buffer 1 eluate HCP (ppm)	307	527	1171
Buffer 2 eluate HCP (ppm)	382	710	1782
Buffer 3 eluate HCP (ppm)	2597	294	3098

**Table 4:** Comparison of resin and membrane mAb purification platforms. Protein A Buffers: Equilibration/wash 1 20 mM sodium phosphate + 150 mM NaCl pH 7.6, wash 2 20 mM sodium phosphate pH 7.0, elution 100 mM glycine pH 3.0. CEX buffers: equilibration/wash 1 50 mM sodium acetate + NaCl pH 4.5, 15 mS/cm, wash 2 20 mM phosphate pH 6.5, Elution 20 mM phosphate + NaCl pH 6.5, 11.7 mS/cm AEX buffers: equilibration/wash 1 25 mM Tris pH 7.5.

Purification step		Resin process 4 min residence time	Membrane process 6 s residence time
Protein A	B&E load	25 g/L	40 g/L
	B&E yield	95%	95%
	Elution HCP	2476 ppm	294 ppm
CEX	B&E load	50 g/L	55 g/L
	B&E yield	80%	85%
	Elution HCP	77 ppm	21 ppm
AEX	FT load	250 g/L	20,000 g/L
	FT yield	99%	99%
	FT HCP	7 ppm	9 ppm

**Table 5:** Evaluation of HCP and aggregate clearance for two processes both employing dual flow though polishing steps.

		Process 1	Process 2
Feed	HCP	247 ppm	1123 ppm
	Aggregates	10.35%	1.91%
HD-Sb membrane column (FT mode)	pH	5.5	7.5
	Load	300 g/L	300 g/L
	Yield	93%	88%
	FT HCP	47 ppm	162 ppm
	FT aggregate	0.49%	0.75%
HD-Q membrane column (FT mode)	pH	7.5	7.5
	Yield	93%	96%
	FT HCP	3 ppm	26 ppm
	FT aggregate	0.42%	0.74%

**Table 6:** Equilibration and elution buffers HD-Sb and HD-Q membranes in 2 flow through processes.

Membrane	Buffer type	Process 1	Process 2
HD-Sb	Equilibration	50 mM sodium acetate + NaCl, pH 5.5, 10 mS/cm	20 mM sodium phosphate + NaCl, pH 7.5, 2 mS/cm
	Elution	25 mM Tris + 1 M NaCl, pH 8.1	25 mM Tris + 1 M NaCl, pH 8.1
HD-Q	Equilibration	25 mM Tris + NaCl, pH 7.5, 5 mS/cm	20 mM sodium phosphate + NaCl, pH 7.5, 2 mS/cm
	Elution	25 mM Tris + 1 M NaCl, pH 8.1	25 mM Tris + 1 M NaCl, pH 8.1
